# The Story of the Insane from Year to Year

**Published:** 1889-09-07

**Authors:** 


### THE STORY OF THE INSANE FROM YEAR TO YEAR.

Asylum Reports.

### NORFOLK COUNTY ASYLUM.

This is one of the oldest asylums in England, and it is nicely situated close to the River Yare. In the original part of the building is still to be seen the arrangement of single-bedded rooms on either side of the galleries; but many improvements have been made of late years to the old asylum, and a large annexe has been built close to it. The beauty of the airing courts is very striking. On January 1, 1888, the asylum contained 684 patients, and on the last day of the year the numbers had gone down to 667, showing a decrease of 17 during the year—a rare occurrence in a county asylum. The County of Norfolk is to be congratulated, too, on having 100 vacant beds. The admissions reached 158, the discharges 93, and the deaths 82. The recoveries reached the very creditable percentage of 44·93, and the death rate was a fraction over 12 per cent. on the average number resident. Phthisis was the cause of death in only six cases, which, in an asylum of this size, must be looked on as decidedly low, and it speaks well for the hygiene of the institution. A very high proportion of the male patients employ themselves, amusements are plentifully provided, and altogether the asylum would seem to be in excellent order. Many minor improvements have been carried out during the year, and, as already said, the sanitary condition of the building is good. The committee record their "satisfaction with the efficient manner in which Dr. Thomson has managed the establishment during the year," and evidently the word of praise is well merited. It is pleasing to notice that the clerk, the head attendant, and the cook were granted retiring pensions. The weekly rate of maintenance stands at 8s. 2d. The medical superintendent's report is admirably arranged.

### BARONY PAROCHIAL ASYLUM, AT LENZIE, NEAR GLASGOW.

Some critics tell us that this is the crack asylum of the kingdom, so far as institutions for pauper lunatics are concerned, and everyone who has visited it comes away with the impression that it is one of the best. At the end of the year 548 patients were in residence, which showed an increase of eight on the previous year, and we gather from the report that the asylum is full. An attempt to relieve the overcrowding has been made by discharging chronic cases to board in private families, but many of these have been returned to the asylum, so that the relief obtained has not been sufficient to obviate the necessity for the further enlargement of the building which the visitors refer to. This boarding-out system is a favourite move in Scotland, and it certainly seems to have met with a fair amount of success in some districts of that country, whereas in England the few trials which have been made have not been so encouraging as to lead to a repetition. The year's admissions amount to 204, the discharges to 155, and the deaths to 41. These numbers yield the following percentages: recoveries 40 per cent. on admissions, and deaths, 5·31 per cent. on the average number resident. The weekly rate of maintenance is 9s. 8¼d., which is greatly above the average of our English county asylums. Of the admissions, 18 were owing to hereditary influence and 27 to drink.

